# Efficacy and safety of anlotinib for the treatment of advanced bone and soft tissue sarcomas: a systematic review and meta-analysis

**DOI:** 10.3389/fonc.2025.1703261

**Published:** 2025-11-28

**Authors:** Zhipeng Li, Peng Fang, Shiwen Shen, Lei Zhang, Rui Xie, Chengjun Li

**Affiliations:** Department of orthopedics, Jinling Hospital, Affiliated Hospital of Medical School, Nanjing University, Nanjing, China

**Keywords:** anlotinib, sarcoma, advanced, targeted therapy, meta - analysis

## Abstract

**Background:**

Sarcoma, a rare and highly heterogeneous malignant neoplasm originating from mesenchymal tissues, is broadly classified into bone sarcoma and soft tissue sarcoma depending on where they occur. Patients with advanced or metastatic sarcomas face a poor prognosis. Conventional chemotherapy regimens demonstrate limited efficacy with substantial adverse effects, and therapeutic options remain scarce for those experiencing chemotherapy failure or intolerance. The development of tyrosine kinase inhibitors has brought the treatment of sarcoma into a new stage. As a multi-target tyrosine kinase inhibitor, anlotinib exerts antitumor effects through dual mechanisms of anti-angiogenesis and direct tumor cell proliferation inhibition. While it has been increasingly reported to treat bone and soft tissue sarcoma with promising efficacy, there has been no systematic analysis of this application.

**Methods:**

PubMed, Embase, the Cochrane Library, Web of Science, Vip (China), Cnki (China), WanFang (China), and SinoMed (China) databases were systematically searched for relevant studies, published from the inception of each database to July 12, 2025, without language restrictions. The primary outcomes included the objective response rate (ORR), disease control rate (DCR), progression-free survival (PFS), overall survival (OS), and adverse events (AEs). These data were extracted and analyzed using STATA 17.0 software.

**Results:**

A total of 16 studies with 787 participants were included in this meta-analysis. In terms of clinical efficacy, the pooled outcomes indicated that ORR and DCR were 8.8% (95%CI: 6.2%–11.7%) and 70.7% (95%CI: 64.8%–76.2%), respectively. Median PFS ranged from 2.7 to 12.4 months, with a pooled result of 6.68 months (95%CI: 5.37–7.98). Median OS ranged from 11.4 to 42 months, with a mean of 19 ± 9.5 months. Furthermore, the 3-, 6-, and 9-month PFS rates were 71.1%, 48.4%, and 32.0%, respectively. The 6- and 12-month OS rates were 85.7% and 67.8%, respectively. With regard to clinical safety, the three most common all-grade treatment-related adverse events associated with anlotinib were hand-foot syndrome (34.7%), hypertension (32.4%), and pharyngalgia (30.6%). However, the incidence of grade 3–4 adverse events was relatively low and manageable; for example, hypertension (7.9%), hand-foot syndrome (2.9%), and pneumothorax (3.0%).

**Conclusions:**

Based on the evidence provided by this meta-analysis, anlotinib demonstrates promising clinical efficacy and a favorable safety profile in patients with advanced bone and soft tissue sarcomas, although additional high-quality clinical studies are required to further evaluate its properties and toxicity.

**Systematic review registration:**

https://www.crd.york.ac.uk/PROSPERO/view/CRD420251103981, PROSPERO.

## Introduction

Sarcomas are a heterogeneous group of mesenchymal malignancies, which can be broadly classified into bone sarcoma and soft tissue sarcoma depending on where they occur (1). They comprise more than 70 histological subtypes with heterogeneous and diverse features in their clinical, pathological, and biological aspects, as well as in their treatment and prognosis (2). Although complete tumor resection with a sufficient surgical-margin is the most effective primary therapy for early sarcomas, 30–50% of sarcomas eventually recur or metastasize after surgery, and some patients present with metastases at the initial diagnosis (3, 4). For patients with advanced, refractory, metastatic, or relapsed bone and soft tissue sarcomas, the prognosis is poor and doxorubicin-based systemic chemotherapy is preferred as first-line treatment (5). However, the five-year survival of chemotherapy ranges between 15 and 30%, with a median overall survival of 1.5–2 years (6). Furthermore, the long-term use of anthracyclines and other cytotoxic drugs is associated with an increased risk of cardiomyopathy (7). Hence, it is urgent to explore new methods to improve long-term disease control and overall survival for advanced bone and soft tissue sarcoma.

As a prerequisite for invasive tumor growth and metastasis, angiogenesis is a key control point in tumor progression (8). Anti-angiogenic therapy was not only an initial strategy in the targeted treatment of sarcoma but also remains its foundation today (9). Vascular endothelial growth factor (VEGF) is a major driver of angiogenesis and plays a crucial role in tumor growth, metastasis, and invasion (10). Besides, dysregulation of the FGF/FGFR signaling pathway facilitates cancer progression and augments the angiogenic potential of the tumor microenvironment (11). In addition, platelet-derived growth factor (PDGF) and c-Kit, critical mediators of cellular proliferation, have emerged as attractive drug targets, offering a potential new strategy for treating advanced bone and soft tissue sarcoma (12).

Anlotinib (AL3818) is a novel, orally available, and highly potent multi-targeted tyrosine kinase inhibitor. It selectively inhibits VEGFR-2/3 and FGFR-1–4 with high affinity, thereby blocking VEGF-driven signaling pathways. Furthermore, anlotinib suppresses tumor proliferation by targeting additional kinases, including PDGFRα/β, c-Kit, Ret, c-FMS, Aurora-B, and DDR (13). Exhibiting promising efficacy and manageable toxicity in various cancers, anlotinib was recommended for several types of solid tumours including advanced soft tissue sarcoma, non-small cell lung cancer, and medullary thyroid carcinoma (14–16).

Although a number of studies have reported the efficacy and safety of anlotinib in the treatment of sarcoma, due to the low incidence of bone and soft tissue sarcoma, most of studies are non-randomized controlled trials with small sample sizes or uncontrolled statistical analysis and inconsistent outcome indicators. Therefore, this meta-analysis aims to comprehensively evaluate the efficacy and safety of anlotinib in the treatment of advanced bone and soft tissue sarcoma, providing more options for clinical treatment.

## Materials and methods

### Search strategy

This systematic review and meta-analysis was conducted following a rigorous experimental protocol that was prospectively registered on the PROSPERO platform (https://www.crd.york.ac.uk/PROSPERO/view/CRD420251103981). A total of eight databases (PubMed, Embase, the Cochrane Library, Web of Science, Vip, Cnki, WanFang, and SinoMed) were systematically searched for relevant studies, published from the inception of each database to July 12, 2025, without language restrictions. We adopted a search strategy combining MeSH and free words, and the main terms used were as follows: “Sarcoma”, “Soft Tissue Sarcoma”, “Epithelioid Sarcoma”, “Spindle Cell Sarcoma”, “Anlotinib”, “AL3818”, “Catequentinib”, and “Anlotinib Hydrochloride”. Finally, a manual search of the reference lists from all included studies was conducted to identify additional relevant studies.

### Study selection

Studies were included if they met the following inclusion criteria: 1) Enrolled patients were histologically diagnosed with bone or soft tissue sarcoma; 2) Oral anlotinib at a dose of 12 mg once daily from days 1 to 14 every 3 weeks was the intervention, whatever previous treatment regimen was; 3) Patients were reported with interested clinical efficiency outcomes, including objective response rate (ORR), disease control rate (DCR), progression-free survival (PFS) and overall survival (OS); 4) All grades of adverse events (AEs) related to the treatment are the primary safety outcomes; 5) All types of clinical research, including prospective clinical trials and retrospective analysis, can be considered.

Studies were excluded if they met any of the following exclusion criteria: 1) Non-eligible publications(e.g.,laboratory research, animal experiments, reviews, meta-analyses,editorial comments,case reports,meeting abstracts or letters); 2) Duplicate publications or with incomplete data; 3) Sample sizes were less than ten cases; 4) Patients received combination medication during the period of anlotinib administration. To avoid selection bias, two reviewers independently identified potentially eligible articles through the predefined eligibility criteria. Any disagreements were resolved through discussion, with a final decision made by a third reviewer.

### Data extraction

Data extraction from the included studies was conducted independently by two authors using a standardized Microsoft Excel spreadsheet. To ensure the reliability of the extracted data, discrepancies were adjudicated by a third author through an independent review and discussion. The information we extracted from included studies was summarized as follows: 1) Article details: first author, publication year, study period, and study type; 2) Participant characteristics: sample size, gender, age, median follow-up, prior therapeutic regimen and histopathological diagnosis; 3) Anlotinib dosing and administration; 4) Clinical efficacy and safety outcomes: ORR, DCR, OS, PFS, and the incidence of AEs related to anlotinib administration by grade.

### Quality assessment

The Methodological Index for Non-randomized Studies (MINORS) was used to evaluate the quality of including non-controlled trials (17). The MINORS instrument comprised 12 items, each scored from 0 to 2. The first eight items were applicable for assessing the methodological quality of all studies, regardless of the presence of a control group. In addition, the methodological quality of the included retrospective studies was assessed using the Joanna Briggs Institute (JBI) Critical Appraisal Checklist for Case Series, which comprises ten items (18). Two investigators independently evaluated the quality and level of evidence for eligible studies, and any discrepancy was resolved through discussion.

### Statistics analysis

All statistical analyses were performed using Stata software (version 17.0; StataCorp, College Station, TX, United States). To synthesize the endpoint data, we conducted a random effect meta-analysis using the metaprop_one command in Stata, a method which accommodates studies with zero event rates (19). The Freeman-Tukey double arcsine transformation was applied to compute the weighted pooled proportions and their 95% confidence intervals (CIs), which were then back-transformed for interpretation and presented using forest plots. Heterogeneity was assessed using Cochran’s Q test and the I^2^ statistic, with a p-value < 0.10 and I^2^ > 50% indicating substantial heterogeneity. Moreover, subgroup analysis was stratified based on tumor type, comparing bone sarcoma with soft tissue sarcoma. Finally, we conducted sensitivity analysis and assessed publication bias using Egger’s test and funnel plots to evaluate the robustness of the pooled results and identify potential bias.

## Result

### Study selection

**Figure 1:**
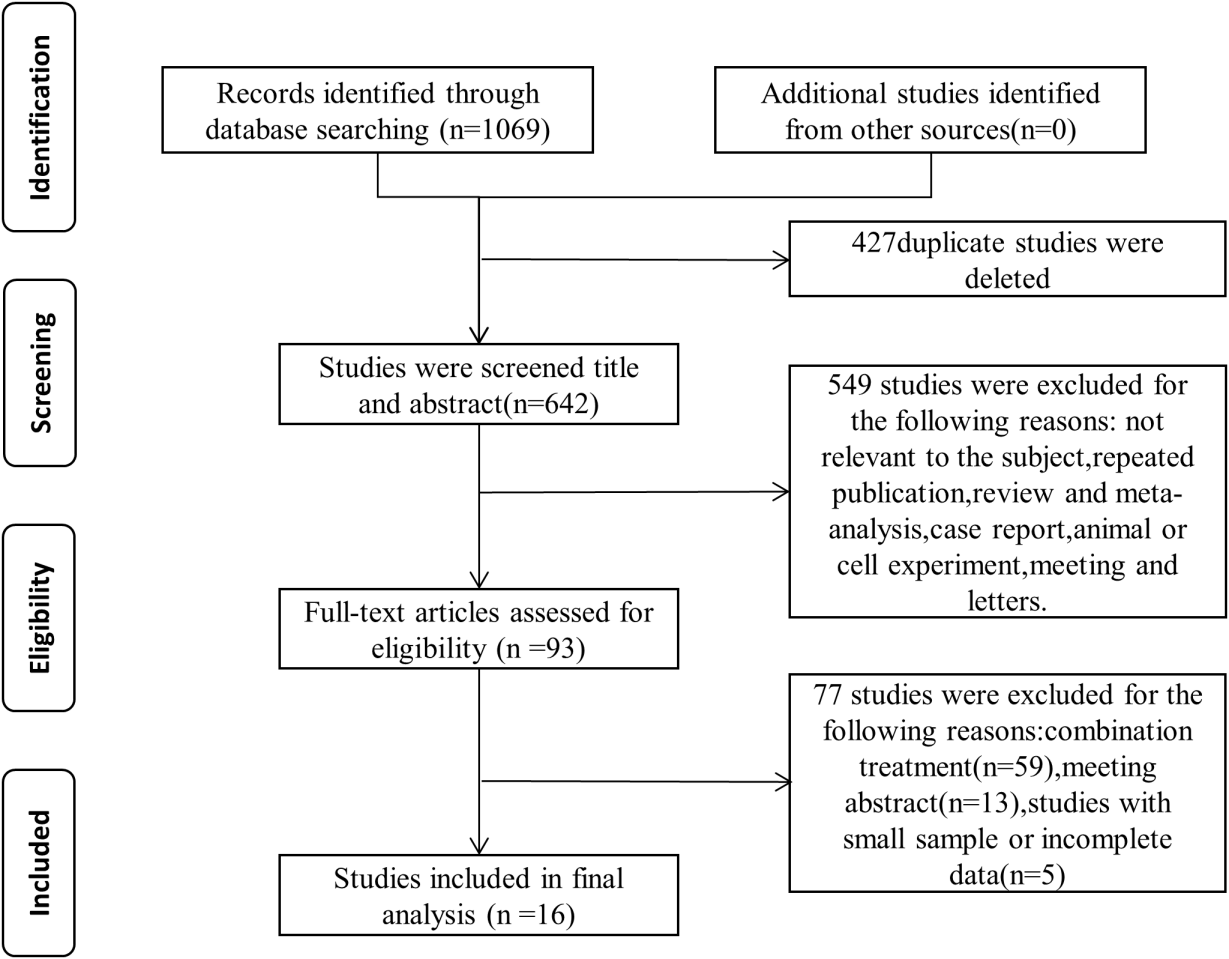
Literature selection flowchart and search yields from individual databases. (PubMed, n=53; EMbase, n=433; Cochrane Library, n=25; Web of Science, n=196; CNKI, n=69; VIP, n=112; WanFang Data, n=106; SinoMed, n=75).

We conducted a comprehensive literature search across eight databases in accordance with the predefined search strategy, obtaining 1069 records. Following the removal of duplicates and initial screening of titles and abstracts, 93 potentially eligible articles were identified. Finally, after full-text review of these articles, a total of sixteen studies (20–35), comprising 787 patients, fulfilled the eligibility criteria and were included in the final meta-analysis. **Figure 1** presents a detailed flowchart illustrating the study selection process.

### Study characteristics and quality assessment

Of the 16 included studies, six were prospective clinical trials and ten were retrospective analysis. Eight studies focused exclusively on soft tissue sarcomas, three solely involved bone sarcomas, and the remaining five included both types. Among soft tissue sarcomas, the three most common pathological subtypes are synovial sarcoma, leiomyosarcoma, and liposarcoma. In contrast, among primary bone sarcomas, the most prevalent types are osteosarcoma, chondrosarcoma, and Ewing’s sarcoma.The total study population comprised 787 patients, with a male-to-female ratio of 1:1.15 and a mean median age of 40.4 years (range, 13–65). All patients received anlotinib monotherapy at 12 mg/day initially, administered in 3-week cycles (2 weeks on, 1 week off). Dose reduction to 10 mg/day or 8 mg/day was implemented for grade 3–4 treatment-related adverse events. Treatment was discontinued due to disease progression, death, or intolerable toxicity. Detailed characteristics of the included studies are summarized in **Table 1**. Additionally, we assessed the methodological quality of the included studies using tools appropriate to each study design. The six non-randomized controlled trials were evaluated with the MINORS instrument, while the ten retrospective case series were appraised using the JBI critical appraisal checklist. Overall, all studies were rated as having moderate to high methodological quality. Detailed results of the quality assessment are presented in **Table 2** and **Table 3**.

**Table 1:** The main characteristics of included studies.

Author/Year	Study design	Study period	Median follow-up months	Sample size	Median age	Histology	Line of therapy	Endpoints
				Male/Female				
Chi,2018 (14)	NCT01878448	2013.5-2015.5	4.2	100/66	45.5	Soft Tissue Sarcoma	Second line	PFS、ORR、DCR、OS、AEs
Li,2024 (21)	NCT03792542	2019.4-2022.6	12.8	24/16	57.5	Soft Tissue Sarcoma	First line	PFS、ORR、DCR、OS、AEs
Liu,2018 (22)	NCT02449343	2015.6-2017.3	23.2*	16/12	39.8	Soft Tissue Sarcoma	Second line	PFS、ORR、DCR、OS、AEs≥3
Xu,2023 (23)	NCT03890068	2019.4-2022.1	17.1	17/32	49	Soft Tissue Sarcoma	Second line	PFS、ORR、DCR、OS、AEs
Tang,2022 (24)	NCT03527888	2018.9-2019.4	9.6	25/17	28	Bone sarcoma	Second or third line	PFS、ORR、DCR、OS、AEs
Lu,2025 (25)	NCT04659733	2020.12-2022.9	16	18/16	13	Bone and Soft Tissue Sarcoma	Second line	PFS、ORR、DCR、OS、AEs
Zheng,2024 (26)	Retrospective	2009.5-2023.5	19.2	19/30	50	Soft Tissue Sarcoma	Second line	PFS、ORR、DCR、OS、AEs≥3
Liu,2021 (27)	Retrospective	2018.6-2020.12	8	29/19	24	Bone sarcoma	First or second line	PFS、ORR、DCR、AEs
Pang,2023 (28)	Retrospective	2018.1-2020.12	46	65/59	48.5	Bone and Soft Tissue Sarcoma	First or second line	PFS、ORR、DCR、OS、AEs
Yan,2023 (29)	Retrospective	2018.6-2021.8	8.2	29/16	43	Bone and Soft Tissue Sarcoma	Second line	PFS、ORR、DCR、AEs
Liu,2021 (30)	Retrospective	2018.5-2019.9	14	12/9	46	Soft Tissue Sarcoma	Second line	PFS、ORR、DCR、AEs
Li,2021 (31)	Retrospective	2018.8-2020.6	8.8	9/8	56	Soft Tissue Sarcoma	First or second line	PFS、ORR、DCR、OS、AEs≥3
Zhang,2022 (32)	Retrospective	2018.6-2020.9	13.3	16/19	65	Soft Tissue Sarcoma	First or second line	PFS、ORR、DCR、OS、AEs≥3
Li,2023 (33)	Retrospective	2018.6-2020.4	10.7	9/6	15.2	Bone sarcoma	Second line	PFS、ORR、DCR、OS、AEs
Gan,2022 (34)	Retrospective	2019.12-2021.6	9	13/19	44	Bone and Soft Tissue Sarcoma	First or second line	PFS、ORR、DCR、AEs
Tian,2020 (35)	Retrospective	2016.5-2019.2	NA	5/8	20.5*	Bone sarcoma	Second line	PFS、ORR、DCR、AEs
				15/14	42*	Soft Tissue Sarcoma	Second line	

NA, not available; *, mean value; PFS, progression-free survival; ORR, objective response rate; DCR, disease control rate; OS, overall survival; AEs, adverse events.

**Table 2:** Quality assessment of included non-randomized controlled trials.

Study	B	C	D	E	F	G	H	Total
Chi,2018 (14)	2	2	2	2	1	2	0	13
Li,2024 (21)	2	2	2	2	1	2	0	13
Liu,2018 (22)	2	2	2	2	1	2	0	13
Xu,2023 (23)	2	2	2	2	1	2	0	13
Tang,2022 (24)	2	2	2	2	1	2	0	13
Lu,2025 (25)	2	2	2	2	1	2	0	13

Items A-H in the MINORS instrument: A, Clear description of aim of the study; B, Inclusion of consecutive patients; C, Prospective collection of data; D, Endpoints appropriate to the aim of the study; E, Unbiased assessment of the study endpoint; F, Follow-up period appropriate to the aim of the study; G, Loss to follow up less than 5%; H, Prospective calculation of the study size.

**Table 3:** Quality assessment of included retrospective studies.

Study	Q1	Q2	Q3	Q4	Q5	Q6	Q7	Q8	Q9	Q10
Zheng,2024 (26)	Y	Y	Y	Y	Y	Y	Y	Y	N	Y
Liu,2021 (27)	Y	Y	Y	Y	Y	Y	Y	Y	N	Y
Pang,2023 (28)	Y	Y	Y	Y	Y	Y	Y	Y	N	Y
Yan,2023 (29)	Y	Y	Y	Y	Y	Y	Y	Y	N	Y
Liu,2021 (30)	Y	Y	Y	Y	Y	Y	Y	Y	N	Y
Li,2021 (31)	Y	Y	Y	Y	Y	Y	Y	Y	N	Y
Zhang,2022 (32)	Y	Y	Y	Y	Y	Y	Y	Y	N	Y
Li,2023 (33)	Y	Y	Y	Y	Y	Y	Y	Y	N	Y
Gan,2022 (34)	Y	Y	Y	Y	Y	Y	Y	Y	N	Y
Tian,2020 (35)	Y	Y	Y	Y	Y	Y	Y	Y	N	Y

Items 1–10 in the JBI Critical Appraisal Checklist for Case Series: Q1, Were there clear criteria for inclusion in the case series? Q2, Was the condition measured in a standard, reliable way for all participants included in the case series? Q3, Were valid methods used for identification of the condition for all participants included in the case series? Q4, Did the case series have consecutive inclusion of participants? Q5, Did the case series have complete inclusion of participants? Q6, Was there clear reporting of the demographics of the participants in the study? Q7, Was there clear reporting of the demographics of the participants in the study? Q8, Were the outcomes or follow up results of cases clearly reported? Q9, Was there clear reporting of the presenting site(s)/clinic(s) demographic information? Q10, Was statistical analysis appropriate?

### Tumor response and survival

**Figure 2:**
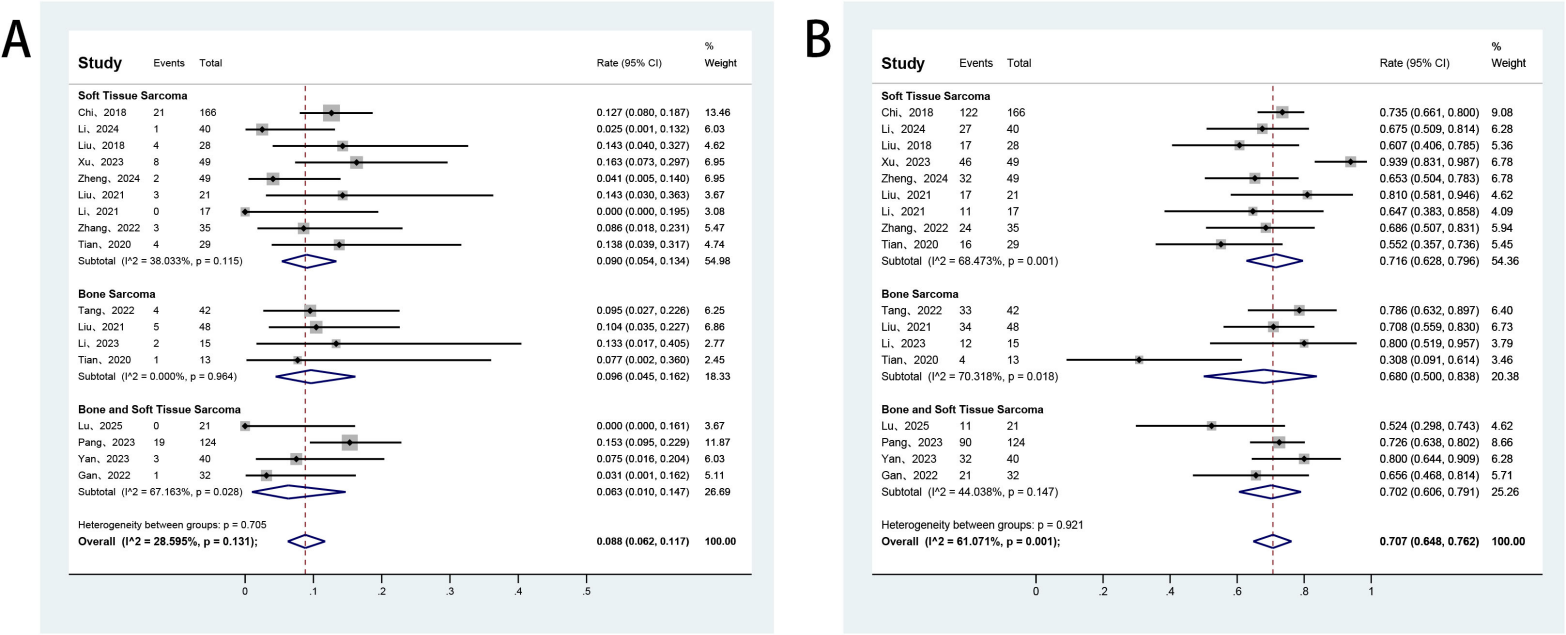
Forest plot of the pooled results for ORR **(A)** and DCR **(B)** across histology subgroups. ORR, objective response rate; DCR, disease control rate.

**Figure 3:**
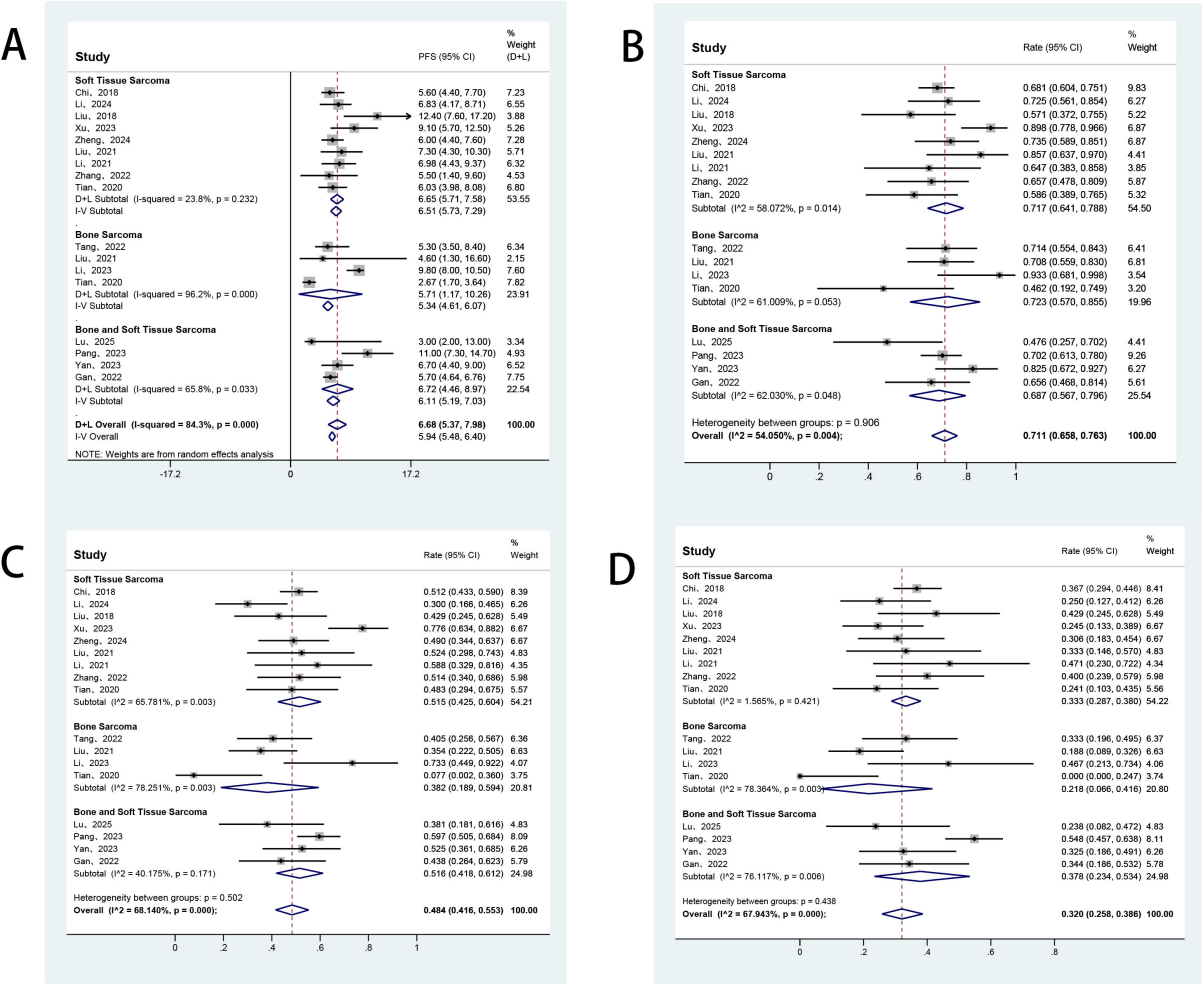
Forest plot of the pooled results for median PFS **(A)** and 3-, 6-, and 9-month PFS rates **(B-D)** across histology subgroups. PFS, progression-free survival.

**Figure 4:**
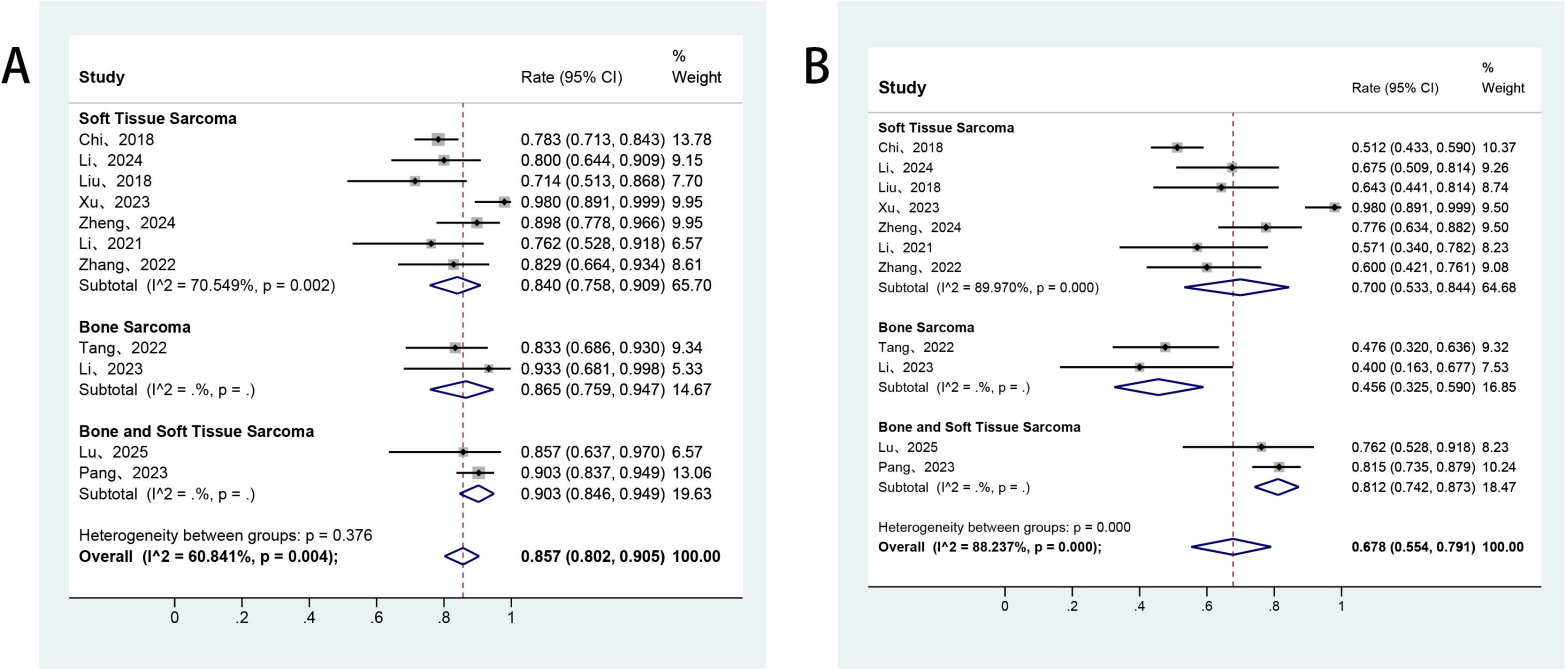
Forest plot of the pooled results for 6- and 12-month OS rates **(A, B)** across histology subgroups. OS, overall survival.

All studies included in this meta-analysis reported the efficacy response of anlotinib in patients with advanced bone and soft tissue sarcomas. Owing to significant heterogeneity among the studies, a random-effects model was applied for the analysis. The pooled ORR was 8.8% (95% CI: 6.2%–11.7%), and DCR was 70.7% (95% CI: 64.8%–76.2%) (**Figure 2**). Additionally, all included studies reported survival outcomes. Among these, 16 studies provided data on PFS, and 11 studies also reported OS. The reported median PFS across studies ranged from 2.7 to 12.4 months. The pooled median PFS was 6.68 months (95% CI: 5.37–7.98), with 3−, 6−, and 9−month PFS rates of 71.1%, 48.4%, and 32.0%, respectively (**Figure 3**). For overall survival, the median OS ranged from 11.4 to 42.0 months, with a mean of 19.0 ± 9.5 months. The pooled 6−month and 12−month OS rates were 85.7% and 67.8%, respectively (**Figure 4**).

### Incidence of adverse events

The severity of adverse events was assessed according to the Common Terminology Criteria for Adverse Events (CTCAE). All included studies reported treatment-related AEs, four of which exclusively reported events of grade≥3. We extracted and pooled the incidence of adverse events to systematically evaluate the safety profile of anlotinib in the treatment of bone and soft tissue sarcomas, with detailed data presented in **Table 4**. The pooled results indicated that the three most commonly reported adverse events were hand-foot syndrome (34.7%, 95% CI: 22.1%–48.3%), hypertension (32.4%, 95% CI: 21.8%–44%), and pharyngalgia (30.6%, 95% CI: 21.5%–40.4%). Diarrhea, fatigue and hypothyroidism were also reported relatively frequently, with incidence rates exceeding 20%. The incidence of grade≥3 AEs was substantially lower, with all reported types occurring in less than 10% of patients, including hypertension (7.9%, 95%CI: 4.7%–11.7%), hand-foot syndrome (2.9%, 95%CI: 1.1%–5.4%), and pneumothorax (3.0%, 95%CI: 1.1% – 5.6%). Furthermore, no permanent morbidity was reported, and all adverse events were effectively managed via drug interruption, dose adjustment, or symptomatic therapy.

**Table 4:** The pooled incidence of adverse events from the included studies.

Adverse events	Pooled incidence (%)	95%CI	Heterogeneity	
			I²(%)	p-value
All grades				
Hand-foot syndrome	34.7	22.1-48.3	91	<0.01
Hypertension	32.4	21.8-44	88	<0.01
Pharyngalgia	30.6	21.5-40.4	65	0.014
Diarrhea	26.5	19.5-34.2	69	<0.01
Cholesterol elevation	26.5	17.3-36.8	76	<0.01
Hypothyroidism	26.2	14-40.5	88	<0.01
TSH elevation	24.6	15.9-34.5	78	<0.01
Fatigue	23.2	16.7-30.5	71	<0.01
Proteinuria	22.6	13.9-32.7	86	<0.01
Stomachache	20.7	10.9-32.5	81	<0.01
Weight loss	19.1	10.9-28.8	56	0.034
Hoarse	18.9	11.2-27.9	72	<0.01
Anorexia	16.9	11-23.7	66	<0.01
ALT elevation	16.8	7.8-28	86	<0.01
AST elevation	15	5.7-27.3	88	<0.01
Hemorrhage	15	8.9-22.3	64	<0.01
Arthralgia	12.8	5-23.1	83	<0.01
Neutropenia	14.6	9.1-21.1	0	0.399
Oral mucositis	11.8	7.3-17.2	39	0.104
Hyperuricemia	11.4	6.7-17.2	0	0.744
Thrombocytopenia	10.4	6.4-15.2	21	0.268
TBIL elevation	10.3	6.5-14.6	0	0.579
Pneumothorax	9.7	5.4-14.8	0	0.546
Nausea	10	4.6-16.9	42	0.125
Dizziness	8.4	4-14	0	0.983
Grade 3-4				
Hypertension	7.9	4.7-11.7	57	<0.01
HFS reaction	2.9	1.1-5.4	34	0.121
Pneumothorax	3	1.1-5.6	18	0.283
Neutropenia	2.4	0.3-5.7	0	0.875
Hemorrhage	2.3	0.3-5.4	0	0.713
Proteinuria	2.1	0.3-4.8	44	0.126
Diarrhea	1.4	0-5.4	37	0.19

TSH, thyroid stimulating hormone; ALT, alanine aminotransferase; AST, aspartate aminotransferase; TBIL, total bilirubin.

### Subgroup analysis

We performed subgroup analyses according to histological subtypes. Among the included studies, nine focused specifically on soft tissue sarcoma(STS) and four on bone sarcoma. The results of subgroup analysis indicated that the ORR and DCR for patients with STS were 9% (95% CI: 5.4%–13.4%) and 71.6% (95% CI: 62.8%–79.6%), respectively, compared to 9.6% (95% CI: 4.5%–16.2%) and 68% (95% CI: 50.0%–83.8%) for those with bone sarcoma. Similarly, survival outcomes were also analyzed in both subgroups. The median PFS was 6.7 months (95% CI: 5.7–7.6) in the STS group and 5.7 months (95% CI: 1.2–10.3) in the bone sarcoma group. The 3-month PFS rate was 71.7% (95% CI: 64.1%–78.8%) for STS and 72.3% (95% CI: 57.0%–85.5%) for bone sarcoma; the 6-month PFS rates were 51.5% (95% CI: 42.5%–60.4%) and 38.2% (95% CI: 18.9%–59.4%), respectively; and the 9-month PFS rate was 33.3% (95% CI: 28.7%–38.0%) for STS and 21.8% (95% CI: 6.6%–41.6%) for bone sarcoma. Additionally, we summarized and compared overall survival (OS) and the incidence of common adverse events between the two subgroups. Detailed results of the subgroup analyses are presented in **Table 5**.

**Table 5:** The pooled results of subgroup analysis.

Endpoints	Soft tissue sarcoma				Bone sarcoma			
	Pooled rate (%)	95%CI	Heterogeneity		Pooled rate (%)	95%CI	Heterogeneity	
			I^2^ (%)	p-value			I^2^ (%)	p-value
Efficacy response								
ORR	9	5.4-13.4	38	0.12	9.6	4.5-16.2	0	0.96
DCR	71.6	62.8-79.6	68.5	<0.01	68	50-83.8	70.3	0.02
mPFS	6.7	5.7-7.6	23.8	0.23	5.7	1.2-10.3	96.2	<0.01
PFS-3	71.7	64.1-78.8	58	0.01	72.3	57-85.5	61	0.05
PFS-6	51.5	42.5-60.4	65.8	<0.01	38.2	18.9-59.4	78.3	<0.01
PFS-9	33.3	28.7-38	1.6	0.42	21.8	6.6-41.6	78.4	<0.01
OS-6	84	75.8-90.9	70.6	<0.01	86.5	75.9-94.7	NA	NA
OS-12	70	53.3-84.4	90	<0.01	45.6	32.5-59	NA	NA
Adverse events								
All grades								
Hypertension	42.2	36.6-47.9	0	0.43	47.7	30.6-65.1	67.6	0.03
Hand-foot syndrome	41.4	29.5-53.9	72.7	<0.01	51.4	39.5-63.2	32.6	0.22
Diarrhea	27.1	17.6-37.7	66.5	0.02	31.5	20.8-43.1	33.8	0.2
Grade≥3								
Hypertension	10.6	5.5-17	63	<0.01	7.9	0.9-18.9	60	0.06
Hand-foot syndrome	2.1	0.2-5.1	34	0.2	6	1.9-11.6	0	0.65
Pneumothorax	2.4	0.3-5.7	26.5	0.25	4.1	0.5-9.7	NA	NA

ORR, objective response rate; DCR, disease control rate; PFS, progression-free survival; OS, overall survival.

### Sensitivity analysis and publication bias

To evaluate the robustness of the meta-analysis, we performed a sensitivity analysis by omitting one study at a time to assess its effect on the pooled results. This analysis revealed that no single study significantly altered the overall pooled results or their 95% CIs, indicating the statistical robustness of the meta-analysis results. Furthermore, publication bias was assessed for the primary outcomes through funnel plots and Egger’s regression test. The results demonstrated no evidence of substantial publication bias, supporting the validity of the meta-analytic conclusions.

### Discussion

Currently, targeted therapy can be employed as either first- or second-line treatment for certain advanced solid tumors. Among these, targeted agents, particularly anti-angiogenic tyrosine kinase inhibitors (AA-TKIs), have demonstrated comparative advantages in terms of individualized treatment and safety profiles, thereby offering novel therapeutic strategies for oncology treatment.

By targeting multiple signaling pathways, including VEGF, fibroblast growth factor (FGF), platelet-derived growth factor (PDGF), FMS-like tyrosine kinase (FLT), rearranged during transfection (RET), and c-Kit, AA-TKIs exert anti-tumor effects via the induction of tumor vascular regression and the suppression of angiogenesis (36, 37). With the advancing understanding of the mechanisms underlying sarcoma pathogenesis, potential targets involved in its pathogenesis are increasingly being identified (38). Furthermore, numerous clinical trials have resulted in the increasing use of AA-TKIs in neoadjuvant or combination therapy regimens for sarcoma patients. For those with advanced sarcoma who are not amenable to surgical resection or are refractory to chemotherapy, AA-TKIs can effectively delay disease progression and convert unresectable bone and soft tissue sarcomas into resectable tumors (39).

Anlotinib is an orally administered multi-targeted TKI that exerts antitumor effects through the simultaneous inhibition of tumor angiogenesis and tumor cell proliferation by selectively targeting FGFR, VEGFR, PDGFR, RET, and c-Kit (40). According to the 2019 Chinese Society of Clinical Oncology (CSCO) guidelines, anlotinib is recommended as a second-line treatment for advanced or refractory soft tissue sarcoma, and may also be used as a first-line therapy for advanced alveolar soft part sarcoma and clear cell sarcoma. A randomized, double-blind, placebo-controlled, multicenter phase IIb trial (ALTER0203, NCT02449343) enrolled 233 patients to evaluate the efficacy of anlotinib in advanced soft tissue sarcoma (14). The results demonstrated that anlotinib significantly improved the median PFS compared with the placebo group (6.27 months vs. 1.47 months), along with higher ORR(10.13% vs. 1.33%) and DCR(55.7% vs. 22.67%). These outcomes are similar to the pooled results from the present meta-analysis, which showed a median PFS of 6.7 months (95% CI 5.7–7.6), an ORR of 9% (95% CI 5.4%–13.4%), and a DCR of 71.6% (95% CI 62.8%–79.6%).

Apatinib is a selective TKI that specifically targets VEGFR2 and demonstrates potent anti-tumor efficacy in a variety of malignancies. It suppresses tumor angiogenesis and induces autophagy and apoptosis in osteosarcoma cells by inhibiting the downstream VEGFR2/STAT3/BCL-2 signaling pathway (41). A retrospective study of apatinib in 105 patients with metastatic osteosarcoma refractory to standard chemotherapy demonstrated an ORR of 37.1%, a DCR of 77.1%, and a median PFS of 4.1 months (42). In a single-arm phase II study (NCT03121846) involving 42 patients with chemotherapy-resistant stage IV soft tissue sarcoma, treatment resulted in a median PFS of 7.9 months, an ORR of 23.7%, and a DCR of 57.9% (43). A meta-analysis was performed on 21 studies involving 827 patients with bone and soft tissue sarcoma who were treated with apatinib (44). The pooled ORR and DCR were 23.9% (95% CI: 18.5–30.2%) and 79.2% (95% CI: 73.8–83.7%), respectively. The median PFS reported across the included studies ranged from 3.5 to 13.1 months.

Regorafenib is an oral multikinase inhibitor targeting VEGFR1–3, c-KIT, RAF, and FGFR, which inhibits tumor angiogenesis and significantly delays tumor growth, and has been approved for treating metastatic colorectal cancer, advanced gastrointestinal stromal tumors, and hepatocellular carcinoma (45, 46). A randomized, double-blind phase II clinical trial of regorafenib for the treatment of advanced soft tissue sarcoma (REGOSARC, NCT01900743) enrolled 182 patients (47). The study population was stratified into four histological subtypes: liposarcoma, leiomyosarcoma synovial sarcoma, and other sarcomas. Results demonstrated that, compared with placebo, regorafenib significantly prolonged median PFS in all subgroups except the liposarcoma cohort. Notably, patients with synovial sarcoma derived the greatest benefit, achieving a median PFS of 5.6 months (95% CI: 1.4–11.6). DUFFAUD et al. (48) conducted a randomized, double-blind, placebo-controlled phase II trial (NCT02389244) of regorafenib in 43 patients with metastatic osteosarcoma. The study met its primary endpoint: 65.4% of patients receiving regorafenib were progression-free at 8 weeks versus 0% in the placebo group. Regorafenib treatment resulted in a median PFS of 4.1 months (95% CI, 2.0 to 6.8), with an ORR of 8% and a DCR of 65.4%. The 3- and 6-month PFS rates were 62% (95% CI, 40 to 77) and 35% (95% CI, 17 to 52), respectively. A meta-analysis involving 179 patients evaluated the safety and efficacy of regorafenib in the treatment of bone sarcoma (49). The results showed that regorafenib treatment was associated with significantly improved 3- and 6-month PFS rates in patients with metastatic or recurrent bone sarcomas compared to controls, with corresponding odds ratios of 2.04 (95% CI: 1.21–2.86; P < 0.01) and 1.03 (95% CI: 0.08–1.99; P < 0.05).

Pazopanib is an oral, selective TKI that targets VEGFR1-3, PDGFR, and c-KIT, resulting in significant inhibition of angiogenesis and tumor cell proliferation. It has been approved for the treatment of advanced renal cell carcinoma and soft tissue sarcoma (50, 51). In a retrospective analysis of 552 patients with metastatic sarcoma treated with pazopanib in Turkey, BILTIC et al. (52) reported that regardless of the line of therapy or histological subtype, the DCR and ORR were 43.1% and 30.8%, respectively. The median PFS was 6.7 months, and overall survival was 13.8 months.A separate retrospective study involving 123 patients evaluated the safety and efficacy of pazopanib in bone and soft tissue sarcomas (53). The results demonstrated a DCR of 46.3%, an ORR of 10.6%, and a median PFS of 3 months.

This meta-analysis included a total of 16 studies comprising 787 patients with bone or soft tissue sarcoma. The pooled ORR, DCR, and median PFS were 8.8% (95% CI: 6.2%–11.7%), 70.7% (95% CI: 64.8%–76.2%), and 6.68 months (95% CI: 5.37–7.98), respectively. In the soft tissue sarcoma subgroup, the ORR, DCR, and mPFS were 9.0% (95% CI: 5.4%–13.4%), 71.6% (95% CI: 62.8%–79.6%), and 6.7 months (95% CI: 5.7–7.6), respectively. Corresponding values in the bone sarcoma subgroup were 9.6% (95% CI: 4.5%–16.2%), 68.0% (95% CI: 50.0%–83.8%), and 5.7 months (95% CI: 1.2–10.3). These results indicate that anlotinib demonstrates comparable or superior clinical efficacy relative to apatinib, regorafenib, and pazopanib for treating these sarcomas. In terms of safety, treatment with anlotinib was associated with adverse events, which represented the most common reason for dose reduction or treatment discontinuation. The three most frequent adverse events were hand-foot syndrome (34.7%, 95% CI: 22.1%–48.3%), hypertension (32.4%, 95% CI: 21.8%–44%), and pharyngalgia (30.6%, 95% CI: 21.5%–40.4%). The incidence of grade ≥3 adverse events was relatively low; the most common included hypertension (7.9%, 95% CI: 4.7%–11.7%), hand-foot syndrome (2.9%; 95% CI: 1.1–5.4%), and pneumothorax (3.0%; 95% CI: 1.1–5.6%). However, all adverse events were manageable through dose interruption, dose reduction, and symptomatic treatment. The pooled safety profile observed in this study is consistent with that of other targeted agents used in the treatment of bone and soft tissue sarcomas. Furthermore, these findings align with previous experience with anlotinib in other refractory solid tumors, including non-small cell lung cancer, thyroid carcinoma, and digestive system neoplasms (54–56).

Finally, we acknowledge that there were several limitations in the present meta-analysis. First, considerable heterogeneity was observed among the included studies. Although subgroup analyses were performed between bone and soft tissue sarcomas, significant heterogeneity persisted across different histological subtypes. Further investigations are warranted to evaluate the efficacy of anlotinib in specific sarcoma subtypes. Second, all incorporated studies were non-randomized and lacked control groups, which inherently lowers the level of evidence and may influence the overall validity of the findings. Moreover, the current analysis assessed only efficacy and safety outcomes without yielding definitive conclusions. Third, this analysis was restricted to studies of anlotinib monotherapy, whereas combination regimens (e.g., with chemotherapy or immunotherapy) are more prevalent in clinical practice. The efficacy and safety of such combinations require further exploration. Fourth, all studies included in this meta-analysis were conducted in Chinese populations with a relatively limited sample size. Whether these findings can be generalized to other ethnic populations remains uncertain. Therefore, large-scale, multicenter, randomized controlled trials are needed to validate the clinical role of anlotinib in comparison to other agents and in diverse patient populations.

## Conclusion

In summary, this meta-analysis demonstrates the efficacy and safety of anlotinib in patients with bone and soft tissue sarcomas, thereby providing evidence to support its clinical application. Treatment with anlotinib was associated with favorable ORR, DCR, PFS, and OS. Although treatment-related adverse events were common, the majority were grade 1–2 and proved largely manageable with appropriate clinical interventions. Therefore, anlotinib is a viable option in both the first- and second-line settings for sarcoma patients. It can be considered for first-line use in patients ineligible for standard chemotherapy, and it is an important option following the failure of prior chemotherapy. Nonetheless, due to the limitations inherent in the included studies, further high-quality clinical trials are required to more definitively establish its activity and toxicity profile within specific single subtype sarcoma.

The progression of precision medicine is shifting cancer treatment toward personalization. Consequently, future clinical trial designs will depend less on histologic subtype and more on specific molecular genetic alterations. This is essential for optimizing targeted therapy in the highly heterogeneous sarcoma family. Furthermore, the limitations of monotherapies have driven the development of combination strategies. Among these, the combination of targeted agents with immune checkpoint inhibitors demonstrates promising synergistic efficacy. A primary objective of next-stage clinical research will be to identify the optimal combination regimens, dosing sequences, and biomarkers for predicting response. Additionally, combining targeted therapy with chemotherapy, radiotherapy, or epigenetic drugs also warrants extensive investigation.

## Data Availability

The original contributions presented in the study are included in the article/supplementary material. Further inquiries can be directed to the corresponding author.
